# Experimental and DFT Investigation of Crystal Violet Biosorption by *Taraxacum officinale* Leaf Biomass

**DOI:** 10.1021/acsomega.6c08045

**Published:** 2026-10-27

**Authors:** Volkan Eyupoglu, Goncagül Serdaroğlu, Osman Çaylak, Muhammed Bora Akın, Serap Çetinkaya

**Affiliations:** † 175171 Çankırı Karatekin University, Faculty of Science, Department of Chemistry, Çankırı 18100, Türkiye; ‡ Sivas Cumhuriyet University, Faculty of Education, Math. and Sci. Edu, Sivas 58140, Turkey; § Vocational School of Health Services, Department of Pharmacy, Cumhuriyet University, Sivas 58140, Türkiye; ∥ Çankırı Karatekin University, Faculty of Engineering, Department of Chemical Engineering, Çankırı 18100, Türkiye; ⊥ Sivas Cumhuriyet University, Faculty of Science, Department of Molecular Biology and Genetics, Sivas 58140, Türkiye

## Abstract

Leaf powder obtained from wild *Taraxacum officinale* was directly used as a biosorbent for the removal of Crystal Violet (CV), a widely used cationic dye, because the leaves are rich in fibers and minerals and have not previously been employed in their untreated form for biosorption. Standard instrumental analyses were employed for the identification of surface functional groups (FT-IR), imaging of surface morphology (FE-SEM), and elemental characterization (EDX). Adsorption was favored under alkaline conditions, reaching a maximum removal efficiency of 46.53% at pH 12, corresponding to an experimental adsorption capacity of 149.1 mg g^–1^, while adsorbent dosages between 100 and 200 mg were effective for dye removal. The untreated biomass surface exhibited adsorption-favoring grooves and stomata. *Taraxacum officinale* biomass exhibited a lignocellulosic structure, with carbon and oxygen contents of 54.26 and 42.46 at%, respectively. Adsorption equilibrium was best described by the nonlinear Langmuir model (*R* ^2^ = 0.948), supporting monolayer adsorption onto a relatively homogeneous surface. The D–R model further supported a physicochemical adsorption mechanism. Frontier molecular orbital (FMO) and natural bond orbital (NBO) analyses were performed at the B3LYP/6-311++G** level in both gas and aqueous media to elucidate the electronic interactions between CV and the lignocellulosic surface.

## Graphical abstract

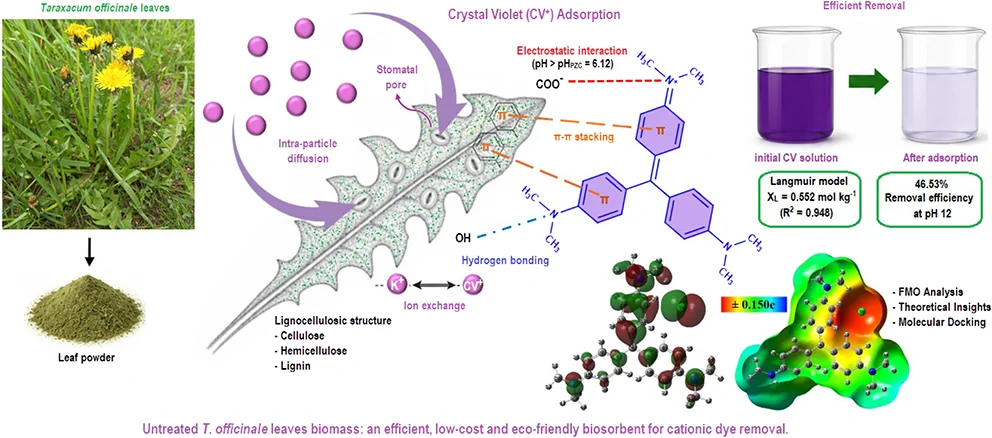

## 1 Introduction

*T. officinale*, dandelion, is an edible perennial plant species of the Asteraceae family and has been renowned since antiquity for its versatile therapeutic properties. The plant has a very large habitat, spanning the new and old continents except Africa. The leaves are a rich source of fiber, minerals, vitamins, and essential oils. All of the organs produce important medicinal phytochemicals, including carotenoids, flavonoids, phenolic acids, as well as lactones, polysaccharides (inulin), sterols, and terpenes. Studies on animal models have discovered that extracts of the plant had important protective consequences for the impaired organs, such as the kidneys and liver, and effects on the strengthening of the immune system, diabetes, and obesity.^1,2^ The plant has also been shown to possess, mainly through the inulin of the root, antibacterial, anticancer, as well as antifungal activities. The leaves produce lactones, phenolic acids, phytosterols, sterols, and triterpenes.^3^

*T. officinale* has recently found interesting applications outside the health realm. Two trials have exemplified its protective action through root extracts against steel corrosion in halogenic^4^ and acidic environments.^5^ In the former, the plant has been annotated as an anodic corrosion inhibitor. Another interesting study has reported that dandelion extracts could produce a lactic acid bacteria-specific biomarker during the course of fermentation by assisting in the instrumental typing of the bacteria of interest.^6^ Its distinct growth pattern in metal-contaminated geographic regions has implied that the plant could also be a biondicator of severely metal-polluted soil.^7^ In this context, biomass-derived materials have gained increasing attention as sustainable and versatile materials for diverse environmental applications.^8,9^

Crystal violet is one of the global dyeing agents. Its use spans from modest research laboratories to industrial mass production plants. Because this chemical is highly noxious in its soluble form in water, through which it has easy access to the biosphere, it has been the focus of entrapment by many laboratory-scale attempts.^10^ This reagent, tris 4-dimethylamino phenyl methylium chloride, is essentially a triarylmethane, C_25_N_3_H_3_OCl.^11^ Being a cationic water-soluble dye with a triphenylmethane structure, it has many deleterious activities on both the aquatic flora and fauna as it directly intervenes with sunlight. Furthermore, upon contact with the skin and through respiration, it prompts various ailments, with the consequences extending up to human cancers.^12,13^

In the past, Meng and coworkers have used CV as a SERS probe to explore the SERS activities of six silver colloids and reported the optimized geometry and Raman spectra of the CV structure at the B3LYP level.^14^ Moreover, Canamares and coworkers have reported that the SERS spectra of the CV structure are governed by the nontotally symmetric vibrations and can be enriched depending on the Herzberg–Teller surface selection rules.^15^ Thus, the charge movement would have an important role in enhancing the SERS by a mechanism that consists of borrowing the intensity via vibrational coupling between the charge-transfer state and the lowest-lying π → π* transition.^15^ In a study on the binary adsorption of methylene blue and crystal violet on mandarin peels, Lima and coworkers have performed the DFT simulations at the B3LYP/6-311++G­(d,p) level to enlighten the removal mechanism of both dyes on mandarin peels.^16^ Moreover, there are reported works supported with computational insights on the CV removal from aqueous solution by adsorption onto different systems, such as the GO (graphene oxide) functionalized with sulfo group,^17^ bentonite clay,^18^ natural plant-derived *Azadirachta indica* bark,^19^ and pits powder/sodium alginate hydrogel beads.^20^ Furthermore, Schäfer and coworkers have investigated the symmetry-breaking effect on the adsorption of CV by using TD-DFT simulations.^21^ The four CV+ structures have been investigated to predict the NLO profiles to provide deep insight into the electronic structure, vibronic, and solvation effects on such octupolar conjugated systems.^22^

Adsorption recipes developed for the entrapment of crystal violet from aquatic sources have often directly used untreated whole plant- or single-cell organisms, while some others have preferred a particular organ or a polymer of a given plant or pretreated plants or their modified forms in alginate beads.^10–13,23^ In our case, a leaf powder obtained from wild *Taraxacum officinale* samples was preferred as the biosorbent, and its potential was assessed with a versatile dyeing agent, Crystal violet.

## 2 Materials and Methods

### 2.1 Chemicals

Organic solvents ethanol, hydrochloric acid, and sodium hydroxide and crystal violet (CV) were of analytical grade and obtained from Merck and Sigma-Aldrich companies.

### 2.2 Preparation of Biosorbent

The plant (Sivas, Turkey) collected in May 2024 was cleansed with distilled water, dried for 48 h at 25 °C, and ground in a mixer.^24^

### 2.3 Experimental Design and Biosorption

Biosorption experiments were carried out using 50 mg of biosorbent and a CV solution with an initial concentration of 300 mg L^–1^ in a total reaction volume of 10 mL. The experiments were performed for 24 h at pH 7.5 and 25 °C. After biosorption, the biosorbent was separated from the liquid phase, and the residual CV concentration in the supernatant was determined spectrophotometrically at 590 nm.^25^ The CV concentration was quantified using a calibration curve relating absorbance at 590 nm to CV concentration. Biosorption was determined from the decrease in CV concentration in the liquid phase by comparing the initial concentration (*C*_i_) with the residual concentration (*C*_f_). The adsorption percentage and adsorption capacity were then calculated using eqs 1 and 2, respectively, whereas the desorption percentage was calculated using eq 3. The calibration curve for the quantitative estimation of the CV dye was prepared at pH 7.5, which corresponds to the optimum pH chosen for the main batch biosorption, kinetics, and isotherm studies. To ensure accurate spectrophotometric measurements and avoid false absorbance readings, special care was taken for samples evaluated at highly alkaline media. Since CV is known to undergo a structural transformation into a colorless carbinol base at pH >9, the supernatant aliquots collected from experiments at pH 10 and 12 were adjusted back to a neutral pH range (∼7.0–7.5) using dilute HCl (0.1 M) prior to the UV–vis measurements. This step ensures that the dye is fully converted back to its colored cationic form for reliable quantification.

1 $$Adsorption\%=\left[\frac{C_{i}-C_{f}}{C_{i}}\right]\times 100$$

2 $$Q=\left[\frac{C_{i}-C_{f}}{m}\right]\times V$$

3 $$Desorption\%=\frac{Q_{des}}{Q_{ads}}\times 100$$

*C*_i_, initial dye concentration (mg L^–1^); *C*_f_, dye concentration (mg L^–1^) at time *t*; *m*, dry biosorbent (g), *V*, reaction volume (L); *Q*_ads_, dye adsorbed (mol kg^–1^), and *Q*_des_, desorbed dye (mol kg^–1^).

#### 2.3.1 Adsorption Isotherms

Adsorption isotherm experiments were performed at initial CV concentrations of 5, 15, 25, 50, 100, 150, 200, 250, 400, and 500 mg L^–1^, while the biosorbent amount (50 mg), initial solution pH (7.5), reaction volume (100 mL), temperature (25 °C), and contact time (24 h) were kept constant. After equilibrium, the biosorbent was separated from the liquid phase, and the residual CV concentration in the supernatant was determined spectrophotometrically at 590 nm. The equilibrium adsorption capacity was calculated from the initial and equilibrium CV concentrations using eq 2. The equations below were used for the isotherm calculations:^26^

4 $$Q=\frac{X_{L}K_{L}C_{e}}{1+K_{L}C_{e}}$$

5 $$Q=K_{F}C_{e}^{\beta }$$

6 $$Q=Q_{\mathrm{DR}}e^{-K_{\mathrm{DR}}\varepsilon^{2}}$$

7 $$\varepsilon =RT\ln\left(1+\frac{1}{C_{e}}\right)$$

8 $$E_{\mathrm{DR}}={\left(2K_{\mathrm{DR}}\right)}^{-0.5}$$

*Q* (mol kg^–1^), adsorbed material B; *K*_L_, isotherm parameters; *C*_e_, the equilibrium concentration (mol L^–1^); *X*_L_ (mol kg^–1^), maximum adsorbent capacity. *K*_F_, Freundlich constant; β, biosorbent surface heterogeneity. *X*_DR_, a measure of biosorption capacity; R, the ideal gas constant (8.314 Jmol^–1^K^–1^); *T*, the absolute temperature (K); *K*_DR_, the activity coefficient (mol^2^KJ^2^); ε, the Polanyi potential.

#### 2.3.2 Calculation of Biosorption Kinetics

Three common models, pseudo-first-order (PFO), pseudo-second-order (PSO), and intraparticle diffusion (IPD), were chosen for the calculation of the biosorption kinetics^26^ (eqs 9–11).

9 $$Q_{t}=Q_{e}\left[1-e^{-k_{1}t}\right]$$

10 $$Q_{t}=\frac{t}{\left[\frac{1}{k_{2}Q_{e}^{2}}\right]+\left[\frac{t}{Q_{e}}\right]}$$

11 $$Q_{t}=k_{i}t^{0.5}$$

*Q*_t_ (mol kg^–1^), adsorbed dye; *t* (time, min); *Q*_e_ (mol kg^–1^), biosorption at equilibrium; *k*_1_, *k*_2_; *k*_i_, the rate constants of the PFO (min^–1^); PSO (mol^–1^ kg min^–1^ IPD (mol^–1^ kg min^–0.5^).

#### 2.3.3 Thermodynamics of Biosorption

Enthalpy (Δ*H*), entropy (Δ*S*), and Gibbs free energy (Δ*G*) were calculated (eqs 12–15).^27^

12 $$K_{D}=\frac{Q}{C_{e}}$$

13 $$\Delta G=‐RT\ln K_{D}$$

14 $$\ln K_{D}=\frac{\Delta S}{R}-\frac{\Delta H}{RT}$$

15 ΔG=ΔH−TΔS

### 2.4 DFT Computational Study

The DFT computations of the CV^+^ ionic and CV–Cl forms were performed at the B3LYP^28,29^ /6-311++G**,^30,31^ level, in the gas and water phase, using the G16W package.^29^ The PCM “Polarized Continuum Model” ^32,33^ was used to simulate the aqueous medium (ε= 78.36) in all DFT computations. The thermochemistry of the systems (CV^+^ and CV–Cl) was evaluated based on the statistical mechanics principles.^34^ GaussView 6.0.16 ^35^ software was used to visualize the optimized geometries and results of the FMOs and MEP analyses.

According to the Koopmans’ theorem,^36^ *I* “ionization energy” and *A* “electron affinity” are obtained from the HOMO and LUMO energies, respectively. Then, the possible reactivity values of the molecular systems can be predicted using the *I* and *A* values defined below:

16 $$I=‐E_{\mathrm{HOMO}}$$

17 $$A=‐E_{\mathrm{LUMO}}$$

18 $$\chi =-\left(\frac{I+A}{2}\right)$$

19 $$\eta =\frac{I-A}{2}$$

20 $$\omega =\frac{\mu^{2}}{2\eta }$$

21 $$\Delta N_{max}=\frac{I+A}{2\;\left(I-A\right)}$$

22 $$\omega^{+}\approx {\left(I+3A\right)}^{2}/\left(16\;\left(I-A\right)\right)$$

23 $$\omega^{-}\approx {\left(3I+A\right)}^{2}/\left(16\;\left(I-A\right)\right)$$

24 $$\Delta \varepsilon_{\mathrm{back}-\mathrm{donation}}=-\frac{\eta }{4}$$

The reactivity indexes are expressed as χ → “electronic chemical potential” η → “global hardness”, ω → “electrophilicity”, Δ*N*_max_ → “the maximum charge transfer capability index”,^37–40^ ω^–^ → “the electrodonating power” and ω^+^ → “the electroaccepting power”,^41^ and ΔE_back‑donat_. → “back-donation energy”.^42^

The second-order-perturbation energy analyses were performed on the NBO basis to determine the intramolecular interactions and their contributions to the decrease in stabilization energy. The *E* ^(2)^ “stabilization energy lowering” is calculated according to the following definitions: *qi* → “the donor orbital occupancy”; *εi* and *εj* → “donor and acceptor orbital energies (diagonal elements)”; *Fij* → “the off-diagonal NBO Fock matrix element”,^43–45^ as given below:

25 $$E^{\left(2\right)}=\Delta E_{ij}=qi\frac{{\left(Fij\right)}^{2}}{\left(\varepsilon j-\varepsilon i\right)}$$

In all DFT computations, the B3LYP hybrid functional, known to provide reliable results, was used in determining the thermochemical and electronic properties, with moderate computational cost and resources.

## 3 Results and Discussion

### 3.1 Characterization

#### 3.1.1 Fourier Transform Infrared Spectroscopy (FTIR)

Functional group composition on the free and CV-occupied *Taraxacum officinale* biomass surface was scanned by FTIR (PerkinElmer 400 FT-IR/FT-FIR Spectrometer Spotlight 400 Imaging System; Waltham, MA, USA) at a resolution range between 4000 and 400 cm^–1^ (Figure 1). The FTIR spectrum of pristine CV was also recorded for direct comparison with the spectra of the biomass before and after biosorption. The pristine CV spectrum exhibited characteristic bands at 1579, 1522, 1355, 1301, 1229, 1161, 1061, 940, 912, 832, and 795 cm^–1^ (Figure 1). The unprocessed biomass powder displayed a characteristic lignocellulose structure. The prominent peak at 3322 cm^–1^, for example, indicated O–H stretching vibrations, produced by hydroxyls of cellulose, hemicellulose, and phenolic compounds in lignin.^46^ The bands at 2988, 2916, and 2850 cm^–1^ corresponded to the aliphatic C–H stretching vibrations of CH_2_– and CH_3_ groups of polysaccharide chains, lipids, or waxy plant constituents. The peak at 1737 cm^–1^ was produced by C=O stretching vibrations of carbonyl groups in ester linkages (such as in pectin or lignin) or carboxylic acids.^47^ The band at 1595 cm^–1^ was attributable to the aromatic C=C stretching vibrations in lignin and/or the asymmetric stretching of carboxylate (COO^–^) groups. The peaks at 1404 cm^–1^ (O–H bending or CH deformation), 1240 cm^–1^ (C–O stretching of aryl ethers in lignin or esters), and 1047 and 1011 cm^–1^ (C–O–C stretching in glycosidic linkages of cellulose/hemicellulose) were produced by the principal plant biopolymer constituents.^48,49^

**Figure 1:**
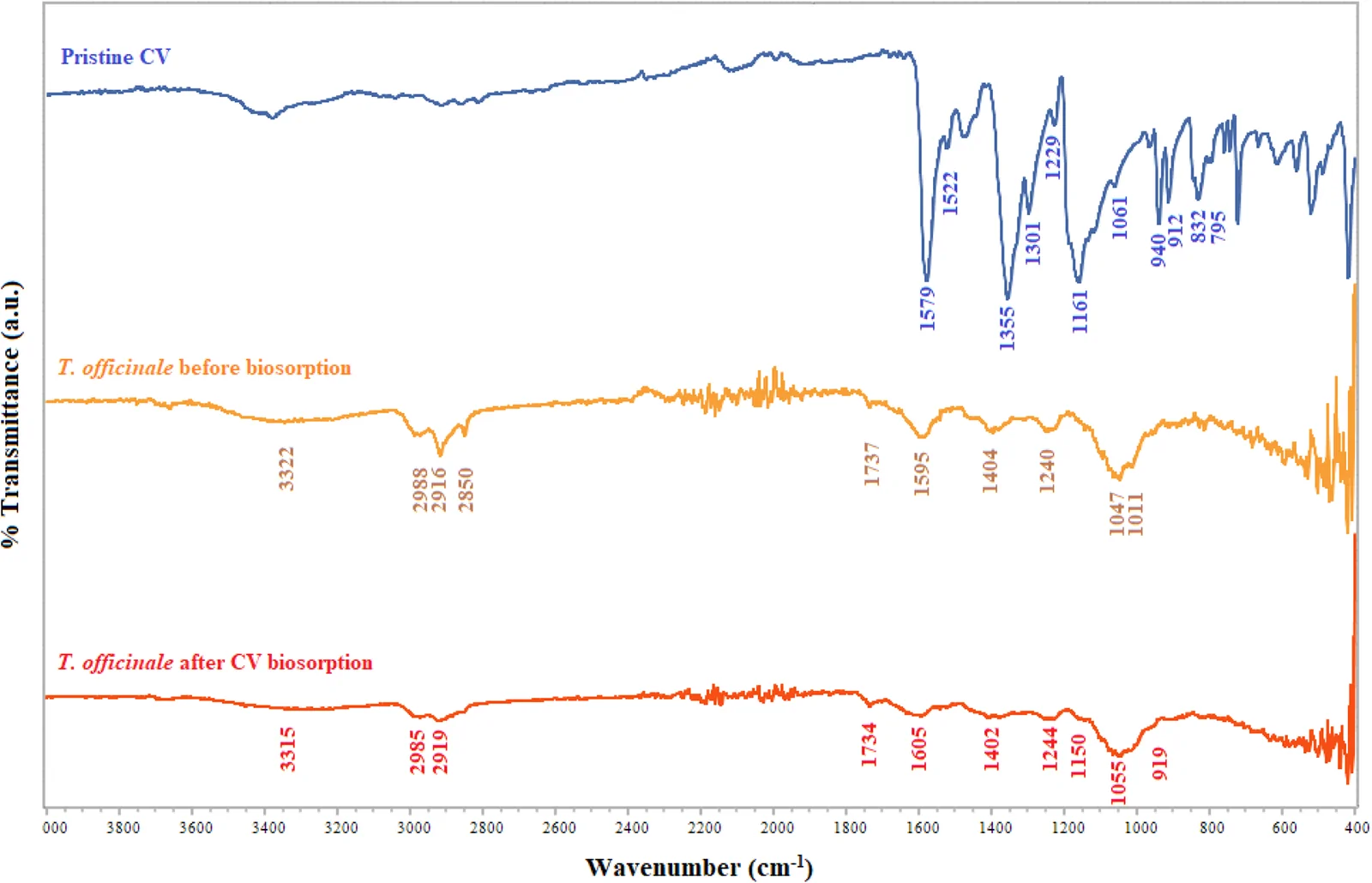
FTIR spectra of pristine CV and *T. officinale* biomass before and after CV biosorption. Spectra are vertically offset for clarity.

The occupied surface moieties displayed adsorption-specific spectra: the O–H stretching peak exhibited a minor shift to 3315 cm^–1^, while the aliphatic C–H peaks (2985 and 2919 cm^–1^) remained rather stable.^23^ The C=O stretching peak at 1734 cm^–1^ displayed a heightened intensity, indicating hydrogen-bonding interactions between CV and carbonyl surface groups (e.g., esters, carboxylic acids). The emergence of a new peak at 1605 cm^–1^, was indicative of aromatic C=C and C=N stretching vibrations specific to the conjugated triarylmethane structure of CV.^50^ This assignment was further supported by the strong band observed at 1579 cm^–1^ in the pristine CV spectrum. This peak therefore provided additional evidence for the presence of bound CV on the biosorbent surface. In addition, the distinct peak at 1150 cm^–1^ was produced by C–N stretching vibrations in CV,^26^ which corresponded closely to the characteristic band at 1161 cm^–1^ observed for pristine CV. The CV-loaded biosorbent also included a shift in the characteristic C–O–C stretching peak, from 1047 cm^–1^ (raw biomass) to 1055 cm^–1^, indicating superposed C–N^+^/C–N vibrational modes of CV. Notably, pristine CV exhibited a strong band at 1061 cm^–1^, supporting the contribution of CV vibrations to the 1055 cm^–1^ band after biosorption. Another polysaccharide-specific band at 1047 cm^–1^ and dye-specific vibrations in the 1050 and 1100 cm^–1^ range, together with the emergence of a new peak at 919 cm^–1^, belonging to one of the aromatic C–H out-of-plane bending modes of CV, also attested that the adsorption reaction was successful. The latter assignment was consistent with the bands at 912 and 940 cm^–1^ in the pristine CV spectrum. Finally, an interaction between the phenolic groups could be represented at 1244 cm^–1^, which was also close to the 1229 cm^–1^ band of pristine CV.

Spectral evidence supporting the effective adsorption could be summarized as follows: (1) CV-associated bands at 1605 cm^–1^ (aromatic C=C/C=N), 1150 cm^–1^ (C–N stretching), and 919 cm^–1^ (aromatic C–H), which showed correspondence with the characteristic pristine CV bands at 1579, 1161, and 912/940 cm^–1^, respectively; (2) the shifted and intensified band at 1055 cm^–1^ (superposition of biomass C–O–C and CV C–N^+^ vibrations), consistent with the 1061 cm^–1^ band of pristine CV; (3) hydrogen bonding involving O–H/C = O groups, represented by the shift to 3315 cm^–1^ and enhanced 1734 cm^–1^ carbonyl stretch; and (4) π–π stacking between the aromatic rings of CV and lignin moieties.

#### 3.1.2 Scanning Electron Microscopy (SEM)

The surface morphology of *Taraxacum officinale* powder was examined before and after adsorption using field-emission scanning electron microscopy (FE-SEM) (Zeiss Gemini SEM 500, Oberkochen, Germany). The elemental composition was ascertained through energy-dispersive X-ray spectroscopy (EDX) in conjunction with a scanning electron microscope (SEM) functioning at a 15 kV accelerating voltage under high-vacuum conditions. SEM micrographs [Figure 2, ×500 (a) and ×2000 (b)] demonstrated significant morphological alterations upon CV adsorption. The vacant biomass surface exhibited grooves and stomata (11.2 ± 2 × 19.9 ± 2 μm; identified using ImageJ), offering inherent adsorption sites. Following adsorption, the formation of an interconnected fibrous network—encompassing stomata—suggested the binding of CV to accessible polysaccharide fibrils.^51,52^ This visual evidence agreed with FTIR data, collectively endorsing a mechanism that integrated surface adsorption, subsurface diffusion through stomata, and fibril-dependent binding.

**Figure 2:**
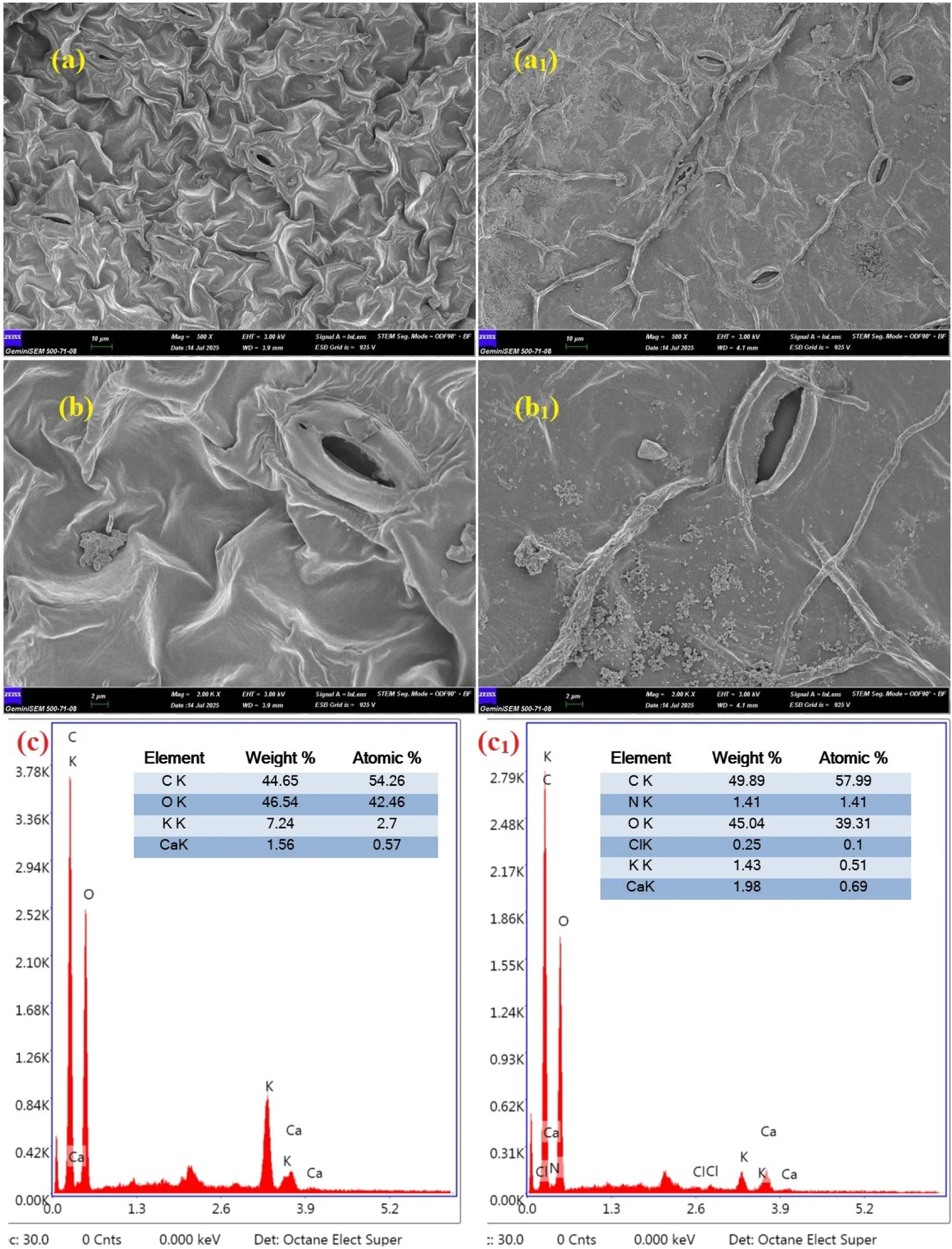
SEM imaging and EDX analysis: (a) untreated surface; (a_1_) CV adsorption at ×500 magnification; (b) untreated surface; (b_1_) CV adsorption at ×2000 magnification; (c) EDX elemental profile; (c_1_) EDX profile after CV treatment.

Energy-dispersive X-ray spectroscopy (EDX) of untreated *T. officinale* biomass (Figure 2c) exhibited a characteristic plant-molecular composition, predominantly comprising carbon (C: 54.26 at. %) and oxygen (O: 42.46 at. %), in alignment with the lignocellulosic structure. Minor inorganic constituents comprised potassium (K: 2.7 at. %) and calcium (Ca: 0.57 at. %), resulting from intrinsic plant electrolytes and ions involved in cell-wall pectin cross-linking.

Following CV biosorption (Figure 2c1), the appearance of nitrogen (1.41 at. %) and chlorine (0.10 at. %), which were absent in the raw biomass, provides additional evidence for the retention of CV on the *T. officinale* surface.^53,54^ A simultaneous pronounced decrease (approximately 81%) in potassium content (from 2.70 to 0.51 at. %) suggests the possible involvement of an ion-exchange mechanism. During adsorption, cationic dye molecules may displace exchangeable mineral cations associated with negatively charged oxygen-containing functional groups on the biomass-derived adsorbents. Similar ion-exchange contributions to CV adsorption have been reported for biomass-derived adsorbents.^55,56^ Although EDX analysis alone cannot conclusively establish an ion-exchange mechanism, the marked decrease in K, together with the appearance of CV-associated N and Cl signals, supports the possible displacement of biomass-associated K^+^ during CV biosorption. The increase in the C/O atomic ratio from 1.28 to 1.48 after CV biosorption, together with the appearance of N and Cl signals, is consistent with the deposition of the carbon-rich CV molecules on the biomass surface.^53^ Although the specific surface area and porosity play a role in traditional adsorbents, the biosorption capacity of raw fungal biomasses is predominantly governed by the abundant surface functional groups (as confirmed by FTIR and DFT analyses) rather than highly developed physical porosity.

### 3.2 pH Effect on the Adsorption

Both the adsorption capacity (*q*_e_) and the percentage removal of Crystal Violet (%Ads) were significantly influenced by the initial reaction pH (Figure 3). At pH 2.0, the removal efficiency was relatively low, only 5.71%. The corresponding adsorption capacity (*q*_e_) was approximately 0.045 mol·kg^–1^. As the pH increased to 8.0, the removal percentage went up to 44.63%, and the *q*_e_ reached approximately 0.351 mol·kg^–1^. A pronounced increase was observed between pH 6.0 and 8.0, where the %Ads rose from 29.26% to 44.63%, and the final dye concentration decreased from 113.32 mg·L^–1^ to 88.70 mg·L^–1^. This corresponded to a sharp increase in *q*_e_, approximately 0.351 mol·kg^–1^. The highest removal efficiencies were achieved at pH 10.0 and 12.0, with %Ads of 45.87% and 46.53%, respectively, and the lowest *C*_e_ values of 86.72 mg·L^–1^ and 85.66 mg·L^–1^. The corresponding *q*_e_ values exceeded 0.36 mol·kg^–1^, indicating enhanced electrostatic attraction between the dye molecules and the negatively charged adsorbent surface at alkaline pH, which is consistent with previous studies reported for cationic dye adsorption systems.^23,26,50^

**Figure 3:**
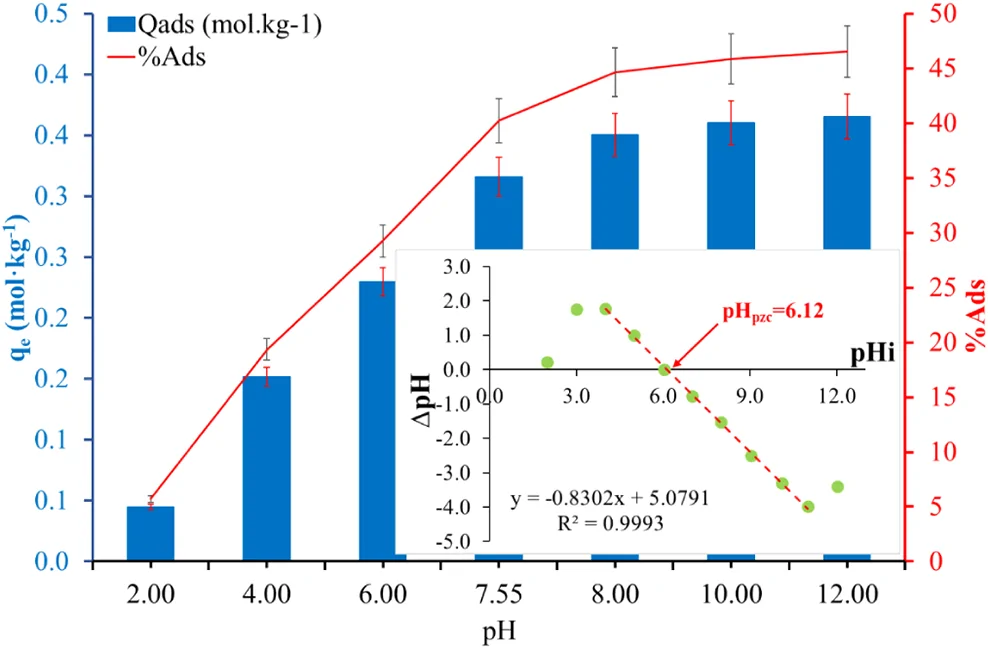
Effect of pH on the adsorption and the PZC of *Taraxacum officinale*.

The point of zero charge (PZC) of the adsorbent was approximately pH_PZC_ = 6.12. Below this point, the surface of the adsorbent was positively charged, leading to electrostatic repulsion with the cationic crystal violet dye. This could explain the lower adsorption performance. Above the PZC, the adsorbent surface became increasingly negative, favored electrostatic attraction, and resulted in higher CV adsorption, which agrees with previously reported adsorption studies for cationic dyes.^13,57^

Although the highest removal was observed at pH 12, such a strongly alkaline condition is not representative of most real wastewater systems and would require an additional chemical adjustment. Therefore, pH 12 was included primarily to elucidate the influence of pH and surface charge on the biosorption mechanism rather than as a recommended operating condition. Considering both the adsorption performance and practical applicability, a more moderate pH of 7.5 was employed in the subsequent biosorption experiments. This pH is also above the determined pH_pzc_, allowing favorable interaction between the negatively charged biosorbent surface and cationic CV without requiring strongly alkaline conditions.

### 3.3 Effect of Adsorbent Dosage

The impact of varying adsorbent dosage on the adsorption performance was studied (Figure 4). Two key parameters were presented: the adsorption capacity at equilibrium (q_e_, mol·kg^–1^) and the percentage removal of dye (%Ads).

**Figure 4:**
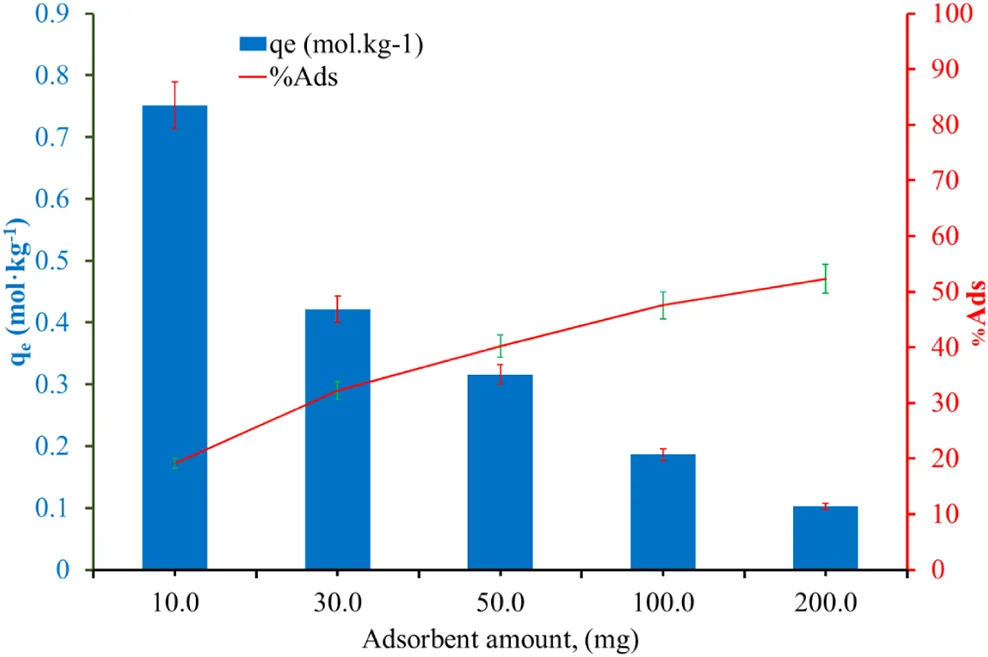
Effect of adsorbent mass.

As the adsorbent mass increased from 10 mg to 200 mg, the %Ads showed a consistent augmentation, rising from 19.15% to 52.28%, indicating improved overall removal efficiency. This was expected due to the high magnitude of available adsorption sites provided by abundant biomass, which is consistent with previous adsorption studies reported in the literature.^13,23,26^ However, the adsorption capacity per unit mass of adsorbent (*q*_e_) exhibited an inverse trend. At 10 mg, *q*_e_ was the highest, reaching 0.752 mol·kg^–1^, but it significantly decreased to 0.103 mol·kg^–1^ at 200 mg. This decrease could be accounted for by surface underutilization caused by particle aggregation. Correspondingly, the equilibrium concentration of dye in solution (*C*_e_) decreased sharply from 129.53 mg·L^–1^ at 10 mg adsorbent to 76.45 mg·L^–1^ at 200 mg.

This dual trend—increasing % removal but decreasing *q*_e_—was typical of adsorption systems and reflected a trade-off between total removal efficiency and sorbent utilization efficiency. The results confirmed that 100–200 mg adsorbent dosages were effective for maximum dye removal, while lower dosages offered higher q_e_ values.

### 3.4 Adsorption Isotherm Modeling

The adsorption equilibrium data were analyzed using four isotherm models: Langmuir, Freundlich, Dubinin–Radushkevich (D–R), and Temkin. Each model was applied in both linear and nonlinear forms to assess the nature of the adsorption process and to identify the best-fitting model. The experimental adsorption data (black triangles) closely followed the predictions of the nonlinear Langmuir model, with minimal deviation across the concentration range (Figure 5, Table 1). The nonlinear Langmuir model exhibited the highest *R* ^2^ value (0.948), confirming its superior performance in describing the equilibrium behavior of CV on the biosorbent surface. Maximum monolayer adsorption capacities (*X*_L_) were calculated as 0.533 mol·kg^–1^ (linear) and 0.552 mol·kg^–1^ (nonlinear). The high values of the Langmuir constant (*K*_L_ = 5760.279 L·kg^–1^ for linear and 4564.786 L·mol^–1^ for nonlinear) also indicated strong affinity between CV and the adsorbent surface. These results suggested that the adsorption occurred predominantly on energetically uniform surface sites, limiting the interactions between adsorbed molecules, which is consistent with previous adsorption studies reported in the literature.^23^

**Figure 5:**
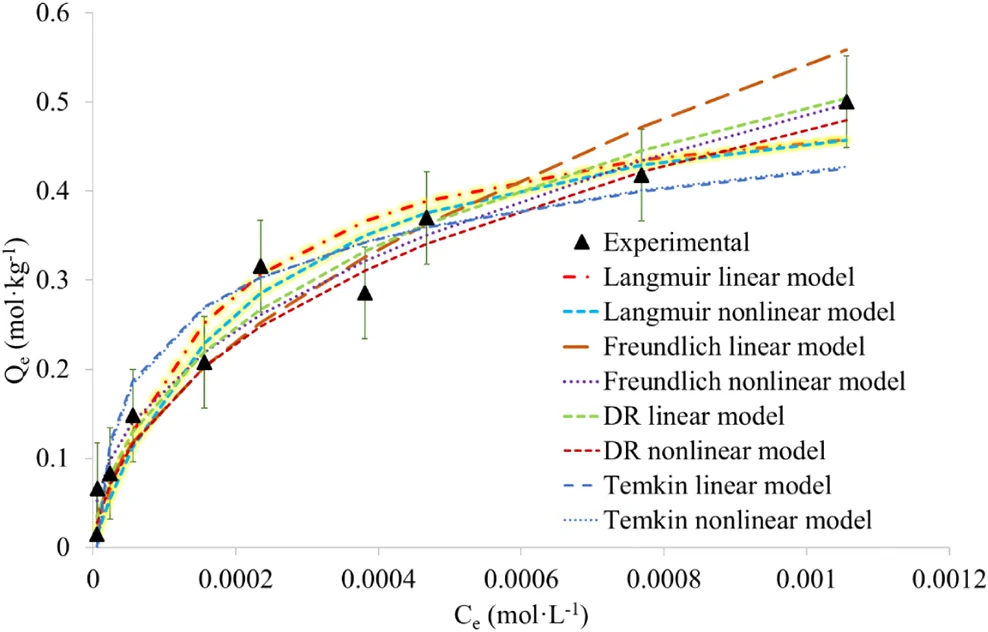
Experimentally obtained adsorption isotherms and their compatibility with those of Langmuir, Freundlich, D-R, and Temkin.

**Table 1:** Langmuir, Freundlich, D-R, and Temkin Isotherms

	Langmuir Parameters		*R* ^2^	Freundlich Parameters		*R* ^2^
**Linear**	*X*_L_ (mol·kg^–1^)	0.533	0.948	β	1.888	0.880
	*K*_L_ (L·kg^–1^)	5760.279		X_F_	21.082	
**Nonlinear**	*X*_L_ (mol·kg^–1^)	0.552	0.948	β	2.325	0.971
	*K*_L_ (L·mol^–1^)	4564.786		X_F_	9.484	

	D-R Parameters		***R*** ^**2**^	Temkin Parameters		***R*** ^**2**^
**Linear**	*K*_DR_ (mol^2^·kJ^–2^) × 10^9^	4.525	0.891	B (J·mol^–1^)	0.081	0.917
	*Q*_DR_ (mol·g^–1^)	1.859		A (L·mol^–1^)	180669.016	
	ε (kJ·mol^–1^)	10.512				
**Nonlinear**	*K*_DR_ (mol^2^·kJ^–2^) × 10^9^	4.700	0.961	B (J·mol^–1^)	0.083	0.896
	*Q*_DR_ (mol·g^–1^)	1.859		A (L·mol^–1^)	164500.353	
	ε (kJ·mol^–1^)	10.314				

The Freundlich isotherm, defining multilayer adsorption on heterogeneous surfaces, also exhibited compatible data. The linear and nonlinear forms yielded *R* ^2^ values of 0.880 and 0.971, respectively. The Freundlich constants (*X*_F_ = 21.082 L·kg^–1^ for linear and 9.484 L·mol^–1^ for nonlinear) and the heterogeneity factor (β = 1.888–2.325) indicated a moderately heterogeneous adsorption surface.^13,50^

The D–R isotherm model, used to distinguish between physical and chemical adsorption, also fitted the experimental data well. Its linear form produced an *R* ^2^ of 0.891, while the nonlinear form gave 0.961. The mean adsorption energy (ε) values were 10.512 kJ·mol^–1^ (linear) and 10.314 kJ·mol^–1^ (nonlinear). The D–R constants (*K*_DR_) were 4.525 × 10^9^ mol^2^·kJ^–2^ (linear) and 4.700 × 10^9^ mol^2^·kJ^–2^ (nonlinear), while the adsorption capacities (*Q*_DR_) were 1.859 mol·g^–1^ (linear) and 1.859 mol·g^–1^ (nonlinear). These values fell within the range between 8 and 16 kJ·mol^–1^ and suggested that the adsorption mechanism involved a combination of physical and chemical interactions rather than purely physisorption or chemisorption. The Temkin isotherm moderately agreed with the experimental data. Its nonlinear form yielded an *R* ^2^ of 0.896, while the linear form was lower at 0.917. The Temkin constant B, related to the heat of adsorption, ranged from 0.081 J·mol^–1^ (linear) to 0.083 J·mol^–1^ (nonlinear), indicating relatively weak interactions. However, the high values of the Temkin constant A (180,669.016 L·mol^–1^ for linear and 164,500.353 L·mol^–1^ for nonlinear) suggested a favorable adsorption potential.

In conclusion, the adsorption of CV was described best by the nonlinear Langmuir model, indicating a monolayer adsorption formed onto a homogeneous surface. The D–R model further supported this by implying a physicochemical adsorption mechanism.

As shown in Table 2, the maximum adsorption capacity obtained for untreated *T. officinale* leaf powder (225.2 mg g^–1^) compares favorably with several recently reported plant-derived biosorbents for CV removal. The capacity obtained in the present study is higher than those reported for raw *Casuarina equisetifolia* cones, untreated *Physalis peruviana calyx*, raw *Saussurea lappa*, natural *Carpobrotus edulis* biomass, and untreated papaya peel, although a higher capacity has been reported for ball-milled royal palm leaf sheath. Importantly, *T. officinale* was used directly without chemical or thermal activation, supporting its potential as a simple and low-cost biosorbent. Nevertheless, direct comparison of adsorption capacities should be interpreted cautiously because experimental conditions, including initial dye concentration, pH, adsorbent dosage, and contact time, differ among studies.

**Table 2:** Comparison of Recent Plant-Derived Biosorbents for Crystal Violet Adsorption

Biosorbent	Preparation	Maximum Adsorption Capacity, qmax (mg g^–1^)	Equilibrium Model	Refs
*Casuarina equisetifolia* cones	Raw biomass	88.94	Freundlich^a^	^57^
*Saussurea lappa* lignocellulosic waste	Raw biomass	33.75	Langmuir	^58^
*Carpobrotus edulis* biomass	Natural, untreated biomass	86.95	Langmuir	^59^
Papaya peel waste	Untreated biomass	99.01	Langmuir	^60^
Royal palm leaf sheath	Ball-milled biomass	454.55	Langmuir	^61^
*Taraxacum officinale* leaf powder	Untreated, directly used biomass	225.2	Nonlinear Langmuir	Present study

a For , Freundlich gave the best overall equilibrium fit, whereas the reported maximum capacity of 88.94 mg g^–1^ was obtained from the Langmuir model.

### 3.5 Effect of Reaction Time

The experimental adsorption kinetics were evaluated using three commonly applied models: pseudo-first-order (PFO), pseudo-second-order (PSO), and the intraparticle diffusion (IPD) model (Figure 6). The pseudo-second-order (PSO) model exhibited the best fit, indicating that the adsorption process was likely governed by chemisorption through sharing or exchange of electrons, which is consistent with previously reported adsorption studies.^26,62^ The calculated equilibrium adsorption capacity (*Q*_e_) of PSO, 0.313 mol·kg^–1^, was in close agreement with the experimental value of 0.316 mol·kg^–1^. This strong correlation supported the validity of the PSO model for describing the adsorption behavior. In comparison, the pseudo-first-order (PFO) model yielded a lower *R* ^2^ value of 0.842, with a *Q*_e_ value of 0.295 mol·kg^–1^. Although the model followed an acceptable trend in the early stages of adsorption, it failed to catch up with the overall kinetic profile at later times. This observation argued that physical diffusion alone might not be the dominant mechanism.^13^

**Figure 6:**
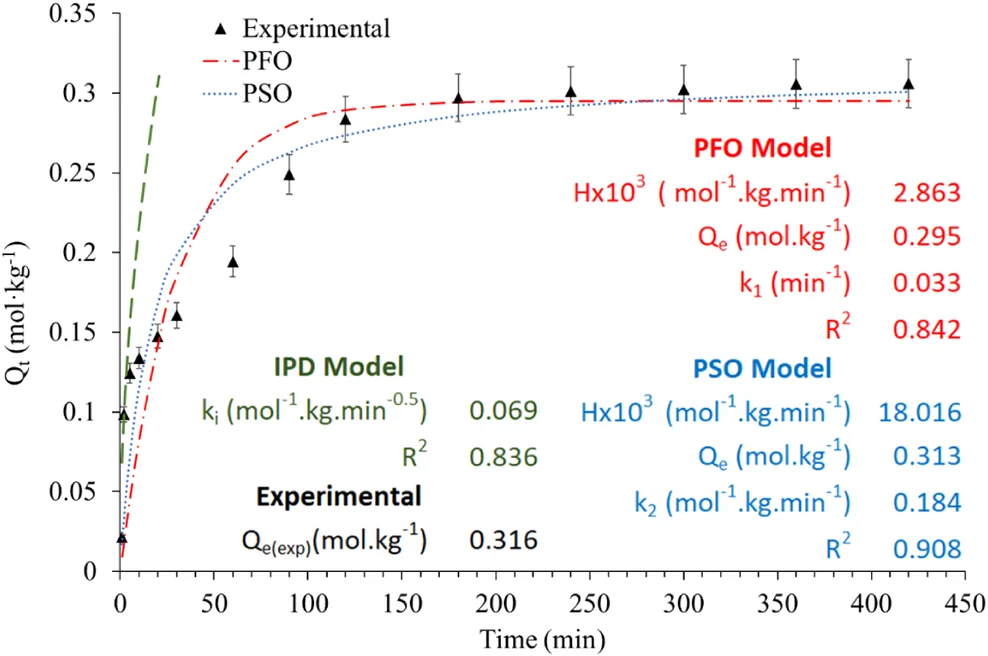
Compatibility of adsorption kinetics with those of PFO, PSO, and IPD algorithms.

The Intraparticle Diffusion (IPD) model showed the poorest fit, with an *R* ^2^ of 0.836. The model parameter *k*_i_ was calculated as 0.069 mol^–1^·kg·min^–0.5^, indicating that intraparticle diffusion also contributed to the adsorption efficiency.^63^

### 3.6 Effect of Temperature

Thermodynamic behavior of the adsorption was elucidated by evaluating the equilibrium distribution constant (*K*_d_) at various temperatures (277.15–333.15 K) (Figure 7). The Van’t Hoff plot of ln *K*_d_ versus 1/T yielded a linear relationship with a high correlation coefficient (*R* ^2^ = 0.9546). This finding indicated the temperature dependence of the adsorption process, following classical thermodynamic theory. From the slope and intercept of the Van’t Hoff equation, the standard enthalpy change (Δ*H*) and standard entropy change (Δ*S*) calculated were +10.49 kJ·mol^–1^ and +121.50 J·mol^–1^·K^–1^, respectively. The positive Δ*H* value indicated that the adsorption was endothermic, favoring greater CV uptake at higher temperatures. The positive Δ*S* reflected increased randomness at the solid–liquid interface, caused by the displacement of water molecules.

**Figure 7:**
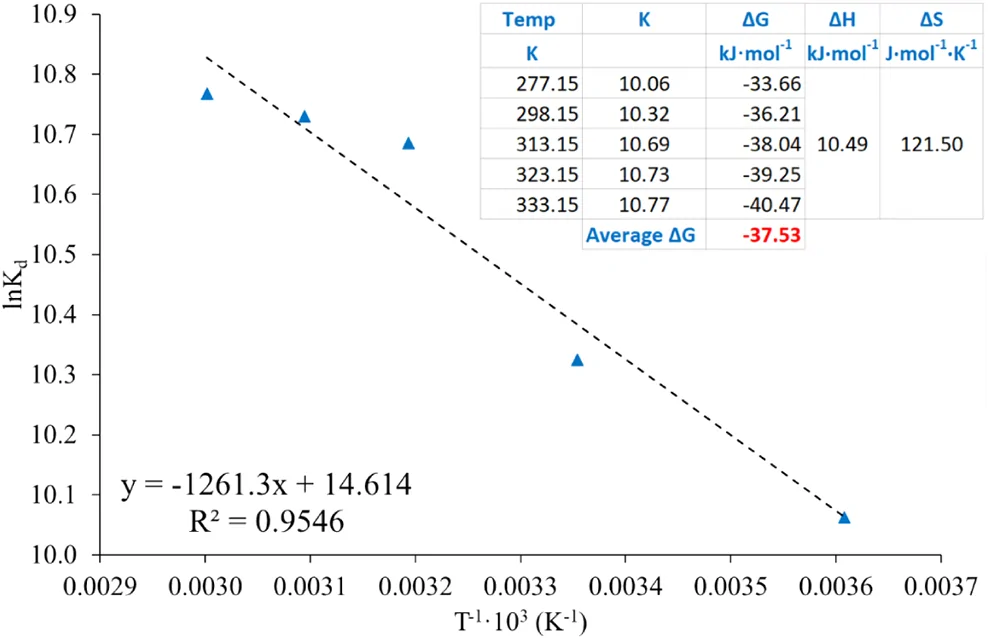
Thermodynamic constants of adsorption.

The standard Gibbs free energy change (Δ*G*) was negative at all the temperature points chosen and ranged from −33.66 to −40.47 kJ·mol^–1^, with an average Δ*G* of −37.53 kJ·mol^–1^, confirming the spontaneous nature of the adsorption process.^64^ Moreover, the magnitude of Δ*G* suggested that the process involved strong interactions, consistent with a physicochemical adsorption mechanism. These findings were thermodynamically consistent with the kinetic and isotherm analyses.

### 3.7 Recovery

Regeneration and reusability of the bioadsorbent were demonstrated using three different desorbents: 0.1 M ethanol (EtOH), 0.1 M NaOH, and 0.1 M HCl (Figure 8). The desorption capacity (*Q*_des_) and corresponding recovery percentages were used to evaluate the effectiveness of each desorbing agent.

**Figure 8:**
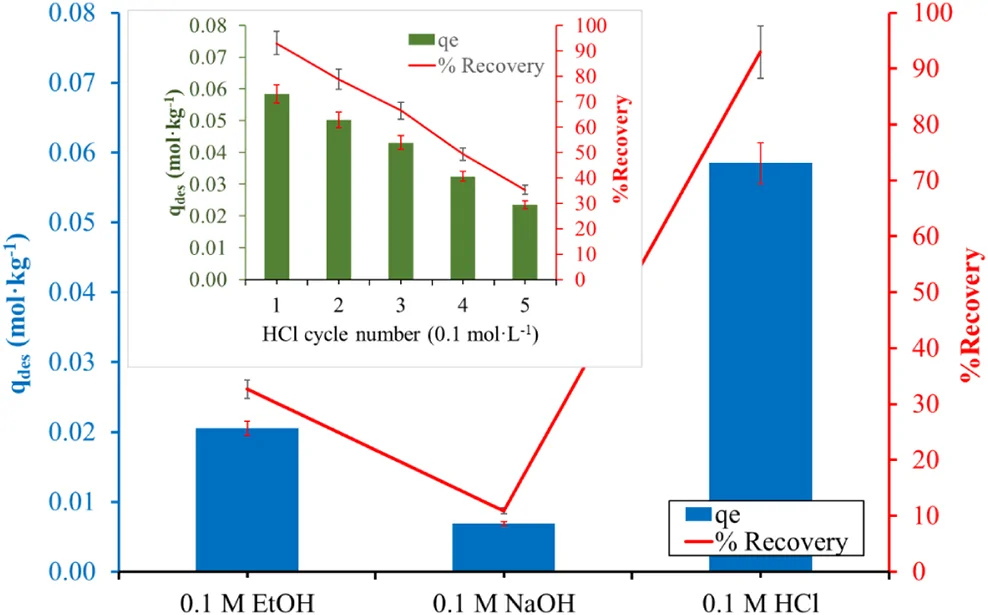
Reusability of the adsorbent.

Among the three desorbents, 0.1 M HCl was the most effective, with a desorption capacity of 5.8451 × 10^–2^ mol kg^–1^ and a recovery efficiency of 92.95%. In contrast, 0.1 M NaOH and 0.1 M EtOH resulted in considerably lower recovery efficiencies of 10.90% and 32.69%, respectively. The high desorption efficiency obtained with HCl can be attributed to the protonation of surface functional groups under acidic conditions, which reduces the negative charge of the biosorbent surface and weakens the electrostatic attraction between the surface and cationic CV molecules. This interpretation is consistent with the experimentally determined pH_pzc_ of 6.12 and with previous studies reporting HCl as an effective eluent for the desorption and recovery of crystal violet from adsorbent materials.^65,66^ Reusability performance was tested over five desorption–adsorption cycles (Figure 8). Although the initial recovery in the first cycle was 92.95%, a gradual decline was observed with each successive cycle. A decrease in adsorption/desorption performance upon repeated regeneration cycles has also been reported for CV-loaded adsorbents.^67^ By the fifth cycle, *Q*_des_ dropped to 2.3547 × 10^–2^ mol kg^–1^, and recovery efficiency decreased to 35.27%. This reduction could be attributed to partial degradation or irreversible occupation of some active sites on the biosorbent surface.

### 3.8 Adsorption Mechanism and DFT Results

The crystal violet adsorption mechanism on *Taraxacum officinale* biomass is elucidated by considering the surface morphology and molecular interactions together (Figure 9). SEM images reveal that the biomass contains porous and stomatal structures, which play a significant role in the transport of dye molecules to the surface and their diffusion into the interior regions. Stomatal spaces, in particular, act as diffusion pathways for CV molecules, which supports the intraparticle diffusion mechanism.

**Figure 9:**
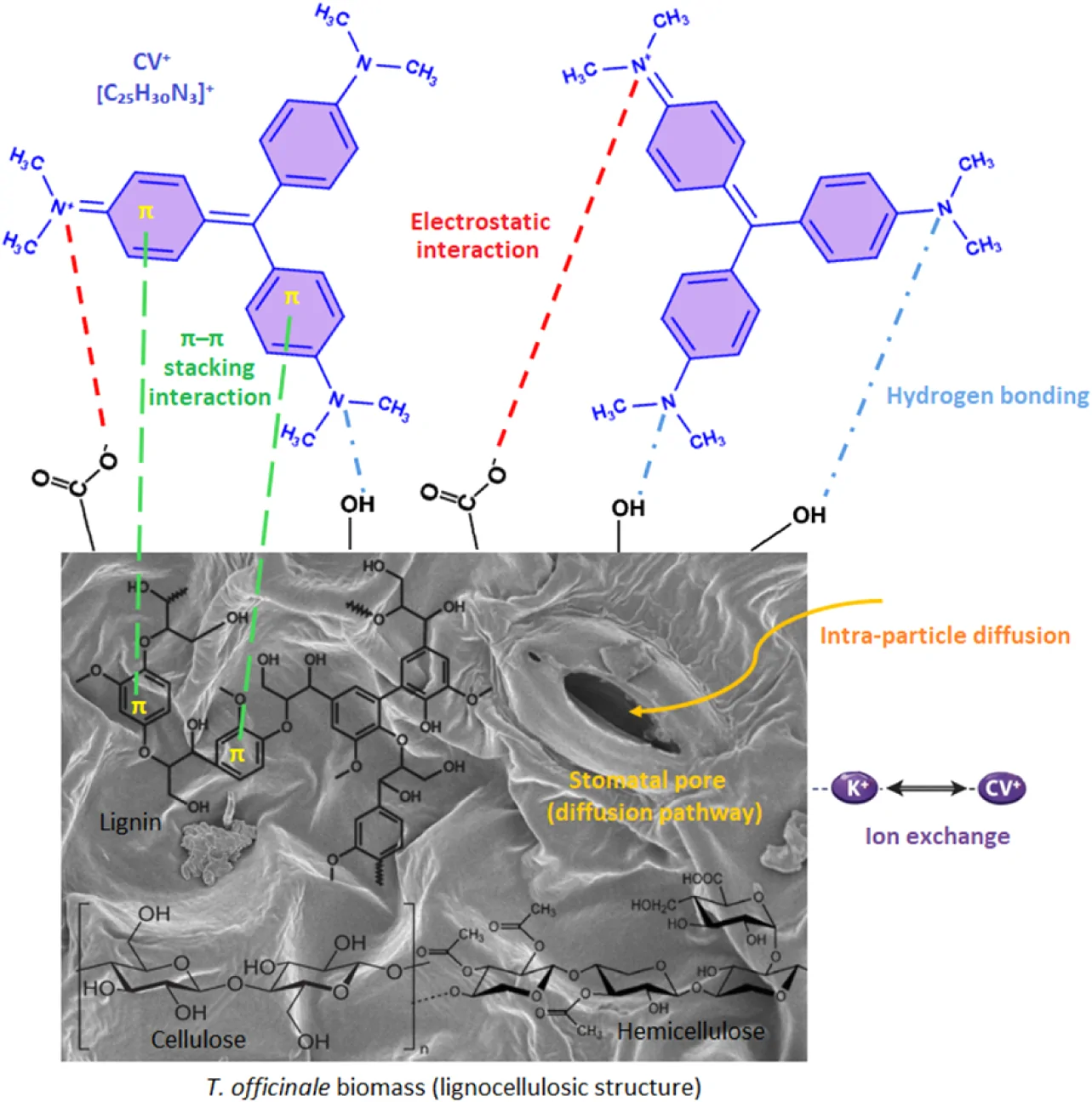
Schematic adsorption mechanism of CV onto *Taraxacum officinale* biomass based on SEM morphology and molecular interactions.

The functional groups of cellulose, hemicellulose, and lignin components in the lignocellulosic structure of the biomass play an active role in the adsorption process. Hydroxyl (−OH), carbonyl (C=O), and carboxylate (−COO^–^) groups, confirmed by FTIR analysis, establish various interactions with the dye. The primary force of adsorption is the electrostatic interaction between the cationic CV^+^ molecule and the negatively charged carboxylate groups on the biomass surface. This is clearly supported by pH effect studies. Specifically, above pH_PZC_ (6.12), the significant increase in CV adsorption as the surface acquires a negative charge confirms that electrostatic attraction is the dominant mechanism.

In addition, hydrogen bonds are formed between the hydroxyl and carbonyl groups and the CV molecule. π–π interactions between the aromatic rings in the lignin structure and the aromatic system of the crystal violet molecule are also an important component of the adsorption mechanism. Furthermore, the decrease in potassium levels observed in EDX analyses indicates that an ion-exchange mechanism occurs between the K^+^ ions on the biomass surface and the CV^+^ ions in the solution.

The optimized geometries and thermochemical data of both the CV^+^ ion and CV–Cl salt forms were given in Figure 10 and Table 3, respectively. Accordingly, the solvent media gain more stability to both CV^+^ and CV forms. Namely, the Δ*G* state function of the CV^+^ ion and CV forms was determined in the gas phase at −1134.626747 and −1595.035451 au, respectively, whereas the free energy of these forms was simulated at −1134.676682 and −1595.104812 au in the water media. From Table 3, the solvation free energies of the CV^+^ and CV forms were calculated at 31.33 and 43.52 kcal/mol; the CV-salt form would gain more stability in the water phase than the CV^+^ ion form. Also, the CV^+^ and CV forms would exhibit more random behavior in the water due to the entropy values increasing from the gas to the water. The dipole moment of the CV^+^ was determined to be almost zero in both media, while the dipole moment of the CV-salt form increased markedly in water due to the presence of the Cl^–^ ion. Moreover, the α index of the CV+ ion and CV–Cl salt was estimated at 461.794 and 491.644 au in gas, which were determined as 689.256 and 707.779 au in water. Herein, the polarizability of both forms would increase with the increase in solvent dielectric constant; the CV–Cl salt would be more polarizable than the CV+, as expected from the existence of the Cl^–^.

**Figure 10:**
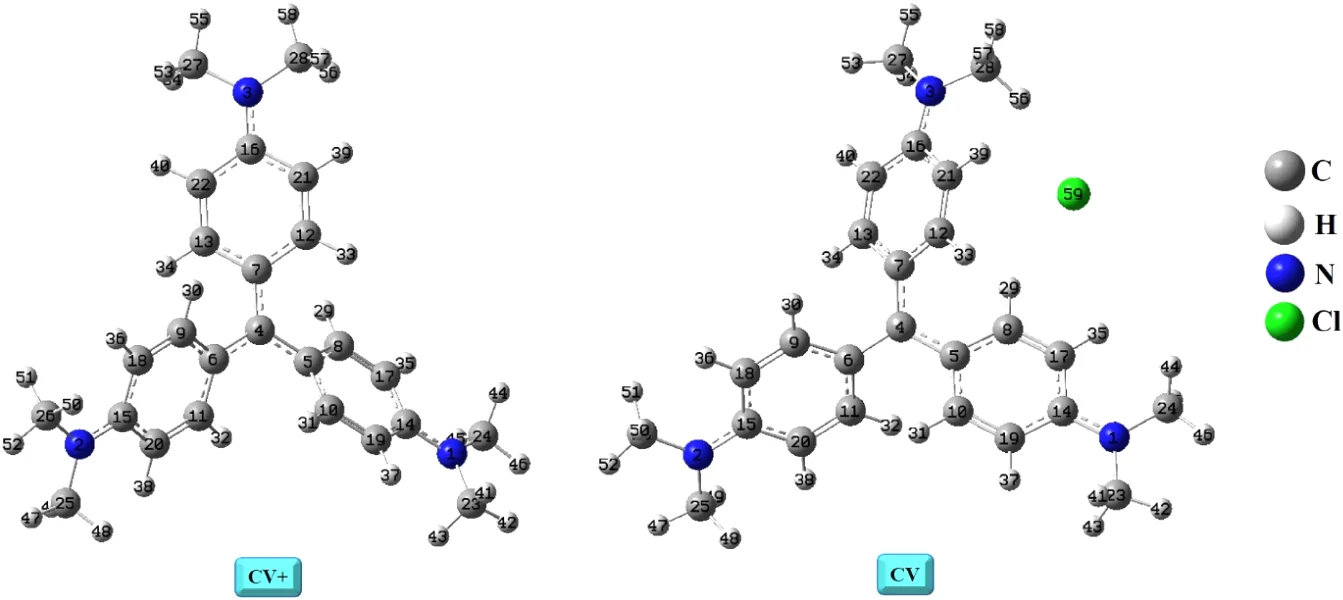
Optimized structures of CV and CV+ molecules.

**Table 3:** Physicochemical Values at B3LYP/6-311++G**

	Gas (ε = 0.0)		Water (ε = 78.39)	
	CV^+^	CV	CV^+^	CV
Δ*E* (au)	–1134.566226	–1594.969543	–1134.615944	–1595.031708
Δ*H* (au)	–1134.537082	–1594.937961	–1134.586734	–1594.999541
Δ*G* (au)	–1134.626747	–1595.035451	–1134.676682	–1595.104812
*E*_therm._ (kcal/mol)	329.943	331.189	329.484	331.203
Cv (cal/(mol K))	106.277	112.166	106.497	112.551
*S* (cal/(mol K))	188.716	205.184	189.311	221.561
μ (D)	0.006	14.293	0.011	36.909
α (au)	461.794	491.644	689.256	707.779
Solvation Energies				
Δ*E*_sol._ (kcal/mol)	31.20	39.01		
Δ*H*_sol._ (kcal/mol)	31.16	38.64		
Δ*G*_sol._ (kcal/mol)	31.33	43.52		

From Table 4, the CV^+^ form (2.706 eV) would be more reactive to the external system than the CV-salt (1.447 eV) form in the gas phase due to the higher energy gap value, as it would be in the water phase. The electrophilicity values implied that the CV^+^ (0.618 eV) would have higher electrophilicity than the CV–Cl form (0.315 eV) in gas, and would have a slightly higher ω index in water. Moreover, the ω+ value of the CV form was determined to be approximately twice that of the CV–Cl form in the gas phase, and also slightly greater than that of the CV–Cl form in water. The Δ*N*_max_ (eV) values of the CV^+^ and CV were determined in the gas as 4.984 and 4.871 eV, whereas they were calculated at 3.289 and 3.275 eV in the water. Overall, the CV^+^ form could interact with the lignocellulosic surface more than the CV–Cl form, depending on the calculated reactivity parameters, especially depicted by the electrophilicity and charge transfer capability indexes.

**Table 4:** Chemical Reactivity Parameters

	Gas		Water	
	CV+	CV	CV+	CV
*H* (−I) (eV)	–8.096	–4.246	–5.769	–5.744
*L* (−A) (eV)	–5.391	–2.800	–3.079	–3.057
Δ*E* (eV)	2.706	1.447	2.690	2.687
χ (eV)	–6.744	–3.523	–4.424	–4.400
η (eV)	1.353	0.723	1.345	1.344
ω (eV)	0.618	0.315	0.267	0.265
ω^+^ (au)	0.500	0.254	0.192	0.190
ω^–^ (au)	0.748	0.383	0.355	0.352
Δ*N*_max_ (eV)	4.984	4.871	3.289	3.275
Δε_back‑donat._ (eV)	–0.338	–0.181	–0.336	–0.336

On the other hand, Figure 11 clearly showed that the CV^+^ ion was covered by a blue color as a marker of the electron-poor profile as a function of the electrostatic potential (V > 0). Thus, the Ns belonging to the CV^+^ ion would interact with the surface via forming H-bonding with the Os atoms. Moreover, the positive charge on the N atom of the CV^+^ structure is delocalized over the structure and would act on the O^–^ ion(s) on the lignocellulosic surface, which would result in a kind of electrostatic interaction. Moreover, the HOMO of the CV^+^ was extended over the phenyl rings (left side), which depicted the possible nucleophilic attack site, while the LUMO expanded over the whole surface as an indicator of the electrophilic attack site(s). On the other hand, the HOMO of the CV–Cl salt expanded over the whole surface except for the −CH_3_ groups on the amine functionalities and Cl^–^ ion, while the LUMO was separated mainly between the Cl^–^ and phenyl ring that was close to Cl^–^ and slightly over the other phenyl rings.

**Figure 11:**
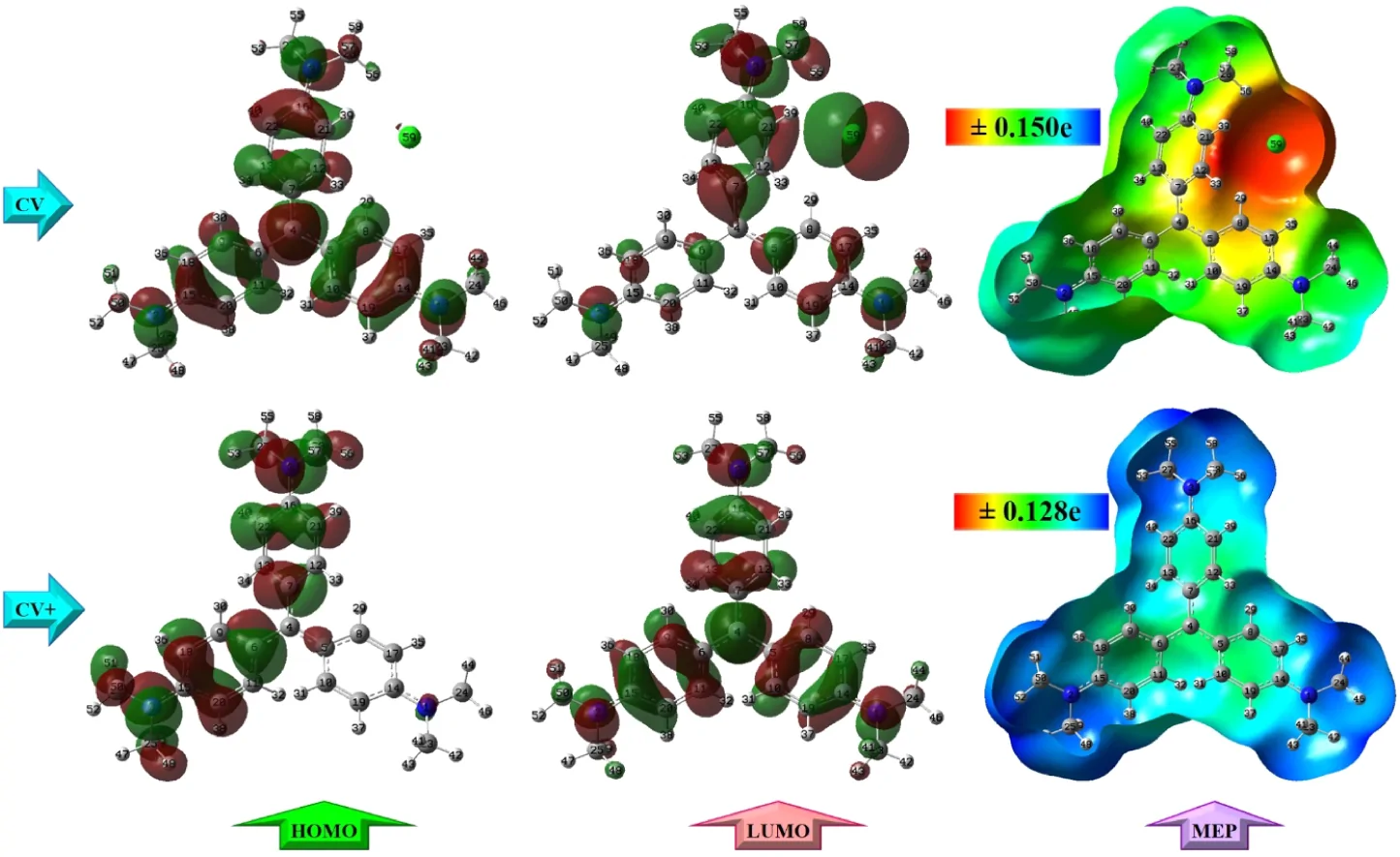
HOMO and LUMO (isoval: 0.02), and MEP (isoval: 0.0004) plots, in gas.

From Table 5, the positive charge on the N atom would be delocalized throughout the structure by the intramolecular charge movement. In fact, the electron deficiency on the N atom would be met with the electron movement, especially π electrons, on the surface of the CV^+^. Namely, the electron movement from the bonding molecular orbitals π C12–C21 (ED_i_ = 1.74553e) and π C13–C22 (ED_i_ = 1.74552e) toward the π* N3–C16 (ED_j_ = 0.63386e) was calculated with the *E* ^(2)^ of 30.66 and 31.16 kcal/mol, respectively. Also, the charge movement from the π C8–C17 (ED_i_ = 1.74552e) and π C10–C19 (ED_i_ = 1.74551e) to π* N1–C14 (ED_j_ = 0.63390e) would imply the strong electron movement on the Cv^+^ surface, due to the high ED_j_ values of these acceptor orbitals. Moreover, the energies of the π C9–C18 → π* N2–C15 and π C11–C20 → π* N2–C15 interactions were computed at 31.11 and 31.19 kcal/mol. From Table 5, the stabilization energy lowering for the charge movement from π N3–C16 to → π* C12–C21 and σ* C22–H40 of the CV^+^ ion was calculated at 5.71 and 9.66 kcal/mol, respectively. Herein, the hyperconjugation (σ → π*) and negative hyperconjugation (π→ σ *) would contribute to the lowering of the stabilization energy. Namely, the charge movement from the σ C28–H56 and σ C28–H58 orbitals to the π* C11–C20 unfilled orbital was determined with the *E* ^(2)^ of 7.35 and 21.98 kcal/mol, respectively. Similarly, the electron delocalization toward unfilled orbitals π* N3–C16, π* C11–C20, and π* C12–C21 from σ C28–H57-related to the −CH3 group on the tertiary amine functionality was estimated with the *E* ^(2)^ of 3.29, 32.25, and 3.81 kcal/mol, respectively. For the CV^+^ ion, the similar σ-conjugations were determined for the other −CH3 related molecular orbitals and neighbor unfilled MOs. Overall, the blue color on the MEP surface of CV+ would be due to the positive charge of the N3 atom belonging to the amine group movement on the whole surface via resonance and hyperconjugative interactions. Also, these interactions provide the CV^+^ ion with a broad surface to interact with the electron-rich region of the related system (lignocellulosic surface).

**Table 5:** NBO Analysis Results of the Selected Compounds, at B3LYP/6-311++G­(d,p) in Gas

Donor(i)	ED_i_/e	Acceptor (j)	ED_j_/e	E^(2)^/kcalmol^–1^	E(j)–E(i)/ a.u	F(i.j)/ a.u
**CV+**						
π N1–C14	1.93104	π* C8–C17	0.26105	4.66	0.37	0.039
		σ* C24–H44	0.01439	7.11	0.34	0.044
π N2–C15	1.93103	π* C9–C18	0.26103	4.65	0.37	0.039
		π* C11–C20	0.26106	5.10	0.34	0.039
π N3–C16	1.93104	π* C12–C21	0.26103	5.71	0.36	0.043
		σ* C22–H40	0.01340	9.66	0.39	0.056
π C8–C17	1.74552	π* N1–C14	0.63390	31.20	0.24	0.085
π C9–C18	1.74553	π* N2–C15	0.63388	31.11	0.24	0.085
π C10–C19	1.74551	π* N1–C14	0.63390	31.22	0.24	0.085
π C11–C20	1.74552	π* N2–C15	0.63388	31.19	0.24	0.085
π C12–C21	1.74553	π* N3–C16	0.63386	30.66	0.24	0.085
π C13–C22	1.74552	π* N3–C16	0.63386	31.16	0.24	0.085
σ C16–C21	1.97058	π* C13–C22	0.26103	65.92	0.19	0.105
σ C16–C22	1.96964	π* C13–C22	0.26103	39.10	0.19	0.081
σ C18–H36	1.97524	π* C11–C20	0.26106	11.44	0.81	0.091
σ C19–H37	1.97570	π* C11–C20	0.26106	9.26	0.83	0.083
σ C20–H38	1.97570	π* C13–C22	0.26103	4.20	0.02	0.008
σ C22–H40	1.97486	π* C11–C20	0.26106	3.37	0.59	0.042
σ C28–H56	1.98879	π* C11–C20	0.26106	7.35	0.50	0.058
σ C28–H57	1.98848	π* N3–C16	0.63386	3.29	0.13	0.022
		π* C11–C20	0.26106	32.25	0.16	0.069
		π* C12–C21	0.26103	3.81	0.19	0.025
σ C28–H58	1.99084	π* C11–C20	0.26106	21.98	0.08	0.041
LP (1) C5	1.08088	π* C8–C17	0.26105	64.05	0.15	0.106
LP (1) C6	1.08087	π* C9–C18	0.26103	63.16	0.15	0.105
		π* C11–C20	0.26106	74.90	0.12	0.102
LP (1) C7	1.08087	π* C12–C21	0.26103	65.48	0.14	0.105
**CV–Cl**						
π C4–C7	1.52384	π* C5–C8	0.43379	8.29	0.27	0.043
		π* C6–C9	0.42238	7.96	0.26	0.041
		π* C12–C21	0.21731	19.94	0.29	0.073
		π* C13–C22	0.25324	25.76	0.27	0.079
π C5–C8	1.53556	π* C4–C7	0.40604	21.87	0.26	0.068
		π* C10–C19	0.28059	22.33	0.27	0.072
		π* C14–C17	0.42478	16.88	0.26	0.060
π C6–C9	1.60027	π* C4–C7	0.40604	16.88	0.28	0.061
		π* C11–C20	0.29541	22.33	0.28	0.072
		π* C15–C18	0.44021	17.62	0.26	0.061
π C10–C19	1.75866	π* C5–C8	0.43379	11.63	0.30	0.056
		π* C14–C17	0.42478	19.47	0.29	0.070
π C11–C20	1.73073	π* C6–C9	0.42238	13.87	0.29	0.059
		π* C15–C18	0.44021	21.52	0.28	0.072
π C12–C21	1.73288	π* C4–C7	0.40604	12.81	0.27	0.055
π C13–C22	1.76808	π* C4–C7	0.40604	12.37	0.29	0.056
π C14–C17	1.54747	π* C5–C8	0.43379	30.28	0.28	0.083
		π* C10–C19	0.28059	13.73	0.28	0.058
π C15–C18	1.58591	π* C6–C9	0.42238	27.59	0.28	0.079
		π* C11–C20	0.29541	13.87	0.29	0.058
LP (1) N1	1.67016	π* C14–C17	0.42478	48.24	0.28	0.106
LP (1) N2	1.69681	π* C15–C18	0.44021	47.74	0.27	0.105
LP (1) N3	1.62403	σ* C24–H44	0.01447	1.65	0.55	0.030
LP (1) Cl	1.99874	σ* C22–H40	0.01358	53.72	0.16	0.082
		σ* C23–H43	0.01659	15.02	0.33	0.063
LP (3) Cl	1.97895	σ* C19–H37	0.01339	21.75	0.39	0.083
		σ* C26–H50	0.01667	27.24	0.43	0.097
LP (4) Cl	1.95751	σ* C26–H50	0.01667	18.79	0.75	0.107

On the other hand, the anomeric interactions (n → σ*) for the CV–Cl salt would be important to the evaluation of the intramolecular charge transfer on the surface, which contributed to decreasing the stabilization energy. Namely, the charge movement from the lone pair of the Cl to the unfilled molecular orbitals σ* C22–H40 and σ* C23–H43 was determined with the *E* ^(2)^ of 53.72 and 15.02 kcal/mol, even though they would have no acceptor orbital occupancy as much as the resonance interactions. The other anomeric interactions for the CV–Cl salt were determined as LP (3) Cl → σ* C19–H37 (21.75 kcal/mol), LP (3) Cl → σ* C26–H50 (27.24 kcal/mol), LP (4) Cl → σ* C26–H50 (18.79 kcal/mol), and LP (1) N3 → σ* C24–H44 (1.65 kcal/mol). Also, the resonances for the CV–Cl structure sourced from the charge movement from the lone pairs of N atoms to antibonding molecular orbitals were determined as LP (1) N1 (ED_i_ = 1.67016e) → π* C14–C17 (ED_j_ = 0.42478e) and LP (1) N2 (ED_i_ = 1.69681e) → π* C15–C18 (ED_j_ = 0.44021e) with the *E* ^(2)^ of 48.24 and 47.74 kcal/mol, respectively.

## 4 Conclusion

This study demonstrated the potential of untreated *Taraxacum officinale* leaf biomass as a low-cost and eco-friendly biosorbent for Crystal Violet (CV) removal from aqueous solutions. CV adsorption was strongly influenced by pH and adsorbent dosage, with enhanced adsorption under alkaline conditions. Equilibrium data were well described by the nonlinear Langmuir model, yielding a maximum adsorption capacity of 0.552 mol kg^–1^ (225.2 mg g^–1^), while the D–R model supported a physicochemical adsorption mechanism. The adsorption kinetics were best represented by the pseudo-second-order model, and thermodynamic analysis indicated that the process was spontaneous and endothermic. Desorption with 0.1 M HCl achieved a recovery efficiency of 92.95% in the first cycle, although recovery decreased upon repeated reuse. The experimental findings, together with the DFT analyses, supported the contribution of electrostatic interactions, hydrogen bonding, π–π interactions, and charge-transfer-related effects to CV adsorption. These findings indicate that untreated *T. officinale* leaf biomass is a promising biosorbent for the removal of cationic dyes from aqueous systems.

## Data Availability

No data were used for the research described in the article.
